# Association of *NCF1* polymorphism with systemic lupus erythematosus and systemic sclerosis but not with ANCA-associated vasculitis in a Japanese population

**DOI:** 10.1038/s41598-019-52920-0

**Published:** 2019-11-08

**Authors:** Nozomi Yokoyama, Aya Kawasaki, Takashi Matsushita, Hiroshi Furukawa, Yuya Kondo, Fumio Hirano, Ken-ei Sada, Isao Matsumoto, Makio Kusaoi, Hirofumi Amano, Shouhei Nagaoka, Keigo Setoguchi, Tatsuo Nagai, Kota Shimada, Shoji Sugii, Atsushi Hashimoto, Toshihiro Matsui, Akira Okamoto, Noriyuki Chiba, Eiichi Suematsu, Shigeru Ohno, Masao Katayama, Kiyoshi Migita, Hajime Kono, Minoru Hasegawa, Shigeto Kobayashi, Hidehiro Yamada, Kenji Nagasaka, Takahiko Sugihara, Kunihiro Yamagata, Shoichi Ozaki, Naoto Tamura, Yoshinari Takasaki, Hiroshi Hashimoto, Hirofumi Makino, Yoshihiro Arimura, Masayoshi Harigai, Shinichi Sato, Takayuki Sumida, Shigeto Tohma, Kazuhiko Takehara, Naoyuki Tsuchiya

**Affiliations:** 1 University of Tsukuba, Faculty of Medicine, Molecular and Genetic Epidemiology Laboratory, Tsukuba, Japan; 2 University of Tsukuba, Graduate School of Comprehensive Human Sciences, Master’s Program in Medical Sciences, Tsukuba, Japan; 3 Kanazawa University, Graduate School of Medical Sciences, Department of Dermatology, Kanazawa, Japan; 4 National Hospital Organization Sagamihara National Hospital, Clinical Research Center for Allergy and Rheumatology, Sagamihara, Japan; 5 National Hospital Organization Tokyo National Hospital, Kiyose, Japan; 6 University of Tsukuba, Faculty of Medicine, Department of Internal Medicine, Tsukuba, Japan; 7 Tokyo Medical and Dental University, Graduate School of Medical and Dental Sciences, Department of Rheumatology, Tokyo, Japan; 8 Tokyo Medical and Dental University, Graduate School of Medical and Dental Sciences, Department of Lifetime Clinical Immunology, Tokyo, Japan; 9 Okayama University, Graduate School of Medicine, Density and Pharmaceutical Sciences, Department of Nephrology, Rheumatology, Endocrinology and Metabolism, Okayama, Japan; 10 Juntendo University, School of Medicine, Department of Internal Medicine and Rheumatology, Tokyo, Japan; 11 Yokohama Minami Kyosai Hospital, Yokohama, Japan; 12 Tokyo Metropolitan Cancer and Infectious Diseases Center Komagome Hospital, Tokyo, Japan; 13 Kitasato University, Department of Rheumatology and Infectious Diseases, Sagamihara, Japan; 14 Tokyo Metropolitan Tama Medical Center, Fuchu, Japan; 15 Himeji Medical Center, National Hospital Organization, Himeji, Japan; 16 Morioka Medical Center, National Hospital Organization, Morioka, Japan; 17 Kyushu Medical Center, National Hospital Organization, Fukuoka, Japan; 18 Yokohama City University Medical Center, Yokohama, Japan; 19 Nagoya Medical Center, National Hospital Organization, Nagoya, Japan; 20 Fukushima Medical University, School of Medicine, Fukushima, Japan; 21 Teikyo University School of Medicine, Department of Internal Medicine, Tokyo, Japan; 22 University of Fukui, Department of Dermatology, Fukui, Japan; 23 Juntendo University Koshigaya Hospital, Koshigaya, Japan; 24 St. Marianna University, School of Medicine, Department of Internal Medicine, Kawasaki, Japan; 25 Center for Rheumatic Diseases, Seirei Yokohama Hospital, Yokohama, Japan; 26 Ome Municipal General Hospital, Ome, Japan; 27 Tokyo Metropolitan Geriatric Hospital, Tokyo, Japan; 28 University of Tsukuba, Faculty of Medicine, Department of Nephrology, Tsukuba, Japan; 29 Juntendo University School of Medicine, Tokyo, Japan; 30 Okayama University Hospital, Okayama, Japan; 31 Kyorin University School of Medicine, First Department of Internal Medicine, Mitaka, Japan; 32 Department of Rheumatology, School of Medicine, Tokyo Women’s Medical University, Tokyo, Japan; 33 The University of Tokyo, Department of Dermatology, Tokyo, Japan

**Keywords:** Genetic association study, Systemic sclerosis, Systemic lupus erythematosus, Vasculitis syndromes

## Abstract

Genome-wide association studies of systemic lupus erythematosus (SLE) in Chinese and Korean populations demonstrated strong association of single nucleotide polymorphisms (SNPs) located in the *GTF*2*I-NCF1* region, rs73366469 (*GTF2I*), rs117026326 (*GTF2I*), rs80346167(*GTF2IRD1*) and rs201802880 (*NCF1*). This region has also been associated with susceptibility to Sjögren syndrome and rheumatoid arthritis; however, association studies with systemic sclerosis (SSc) and ANCA-associated vasculitis (AAV) have not been reported. Here we made an attempt to confirm their associations with SLE in the Japanese population, to find the primarily associated SNP, and to investigate whether these SNPs are also associated with susceptibility to SSc and AAV. By genotyping these four SNPs on 842 SLE, 467 SSc, 477 AAV patients and 934 healthy controls, striking association was confirmed in Japanese SLE. In addition, these SNPs were significantly associated with susceptibility to SSc, but not with AAV. Conditional logistic regression analysis revealed that the association of *NCF1* rs201802880, a missense SNP encoding p.Arg90His, can account for the association of other SNPs by linkage disequilibrium. These results suggested that *GTF2I-NCF1* region is associated with susceptibility to multiple autoimmune rheumatic diseases but not with AAV, and the primarily associated variant may be the missense SNP in *NCF1*.

## Introduction

Autoimmune diseases are caused by a combination of multiple genetic and environmental factors, but the precise mechanisms of their development are largely unestablished. Genome wide association study (GWAS) is an efficient approach to identify the genetic factors of such complex disorders. GWAS of autoimmune rheumatic diseases including rheumatoid arthritis (RA), systemic lupus erythematosus (SLE), systemic sclerosis (SSc) and ANCA-associated vasculitis (AAV) unanimously demonstrated that the strongest association signal is present within the major histocompatibility complex (MHC)^1^ until 2013, when GWAS of Sjögren’s syndrome (SS) in the Chinese population surprisingly demonstrated striking associations of single nucleotide polymorphisms (SNPs), rs73366469 (T > C), rs117026326 (C > T) and rs80346167 (G > A), in a region encoding general transcription factors *GTF2I* and *GTF2IRD1*, which were even stronger than that of the *MHC* region^2^.

Subsequently, Immunochip and replication studies in Chinese and Korean populations also demonstrated strong association of the SNPs located at *GTF2I* region with susceptibility to SLE^3^. Furthermore, this region was also reported to be associated with susceptibility to RA in Korean and Japanese populations^4^. This region has also been shown to be associated with susceptibility to SLE in European American populations, albeit more weakly^5^. Thus, the region appears to be one of the strongest genetic factors for multiple autoimmune rheumatic diseases in East Asian populations.

*GTF2I* encodes general transcription factor II-I (TFII-I). TFII-I usually localizes in the cytoplasm. It is translocated into the nucleus after activation by growth factors, B cell and T cell receptor triggering factors, and endoplasmic reticulum stress. In the nucleus, TFII-I binds to promoter regions of target genes and promotes transcription^6^. In addition, cytoplasmic TFII-I regulates surface expression of Ca^2+^ channel protein TRPC3^6^. Thus, TFII-I has relevant functions to autoimmune diseases.

On the other hand, *NCF1* gene encoding neutrophil cytoplasmic factor 1, a subunit of NADPH oxidase, is one of the responsible genes for chronic granulomatous disease, and is located close to *GTF2I* and *GTF2IRD1* genes. A naturally occurring reduction-of-function polymorphism of *Ncf1* has been positionally identified to be associated with severity of pristane-induced arthritis in rats^7^. Subsequently, introduction of *Ncf1* mutation in mice has been shown to be associated with arthritis, autoimmune encephalomyelitis^8^, and also lupus-like phenotypes with glomerulonephritis and type I interferon signature^9^. In humans, a missense variant (p.Arg90His, rs201802880) in *NCF1*, leading to reduction-of-function of NADPH oxidase, has also been associated with susceptibility to SLE. The *NCF1* and *GTF2I* region variants are in linkage disequilibrium (LD), and two studies strongly suggested that the causative variant of this region is the *NCF1* missense variant^10,11^. However, because of the complicated genomic configuration of this region with the presence of *NCF1* copy number variation (CNV) and highly homologous pseudogenes (*NCF1B* and *NCF1C*), further studies from various populations will be informative in establishing the genetic contribution of each variant of this chromosomal region.

SLE and SSc are both characterized by antinuclear antibodies, and a small proportion of patients exhibit symptoms of both diseases (SSc-SLE overlap syndrome). In a recent cohort study of SSc in Toronto, the prevalence of SSc-SLE overlap was 6.8%^12^. Similarly, although rare, co-occurrence of SLE and AAV has been reported especially in MPO-ANCA positive AAV, and a concept of SLE-AAV overlap syndrome has been proposed^13^. Such co-occurrence suggests the presence of shared genetic factors. With respect to the overlap of susceptibility alleles, out of 116 non-*HLA* loci associated with SLE with P < 5 × 10^−8^ in a large-scale Immunochip analysis (based on the summary statistics downloaded from the NHGRI-EBI GWAS Catalog^14^ for study^5^ downloaded on 07/23/2019) and 18 confirmed SSc susceptibility loci^15^, 10 loci were shared by SLE and SSc. As for AAV, only three loci (*PTPN22*, *PRTN3*, *SERPINA1*) have been confirmed as susceptibility loci except for *HLA*, among which only *PTPN22* is shared with SLE^16,17^. Thus, a rather small proportion of SLE susceptibility loci appear to be shared with SSc and AAV. To distinguish the susceptibility loci shared by multiple autoimmune rheumatic diseases and those specific for each disease will eventually lead us to deeper understanding of pathogenesis of these diseases.

Although *GTF2I-NCF1* region associations have been reported in SLE, SS and RA, association studies have not been reported for SSc and AAV. In addition, to our knowledge, association study between this region and SLE has not been reported in the Japanese population. In this study, we examined whether the SNPs in *GTF2I-NCF1* region are associated with susceptibility to SSc and AAV in addition to SLE. We also made an attempt to identify which SNP plays the primary role among these four SNPs.

## Results

### Association of *GTF2I-NCF1* region SNPs with overall SLE and SSc

First, we examined whether the *GTF2I-NCF1* region SNPs are also associated with susceptibility to SLE in the Japanese population. The previously reported risk alleles at the four SNPs were strikingly increased in patients with SLE in comparison with healthy controls also in the Japanese population (Table 1).

**Table 1:** Associations between the SNPs and SLE, SSc and AAV under the additive model (case-control analysis).

	n	rs73366469 (T > C) *GTF2I-GTF2IRD1*			rs117026326 (C > T) *GTF2I*			rs80346167 (G > A) *GTF2IRD1*			rs201802880 (G > A) *NCF1*		
		MAF (%)	P_uncorr_ (Q)	OR (95%CI)	MAF (%)	P_uncorr_ (Q)	OR (95%CI)	MAF (%)	P_uncorr_ (Q)	OR (95%CI)	MAF (%)	P_uncorr_ (Q)	OR (95%CI)
SLE all	842 (826)	300 (17.8)	8.47 × 10^−14^ (6.22 × 10^−13^)	2.36 (1.89–2.96)	298 (17.7)	7.96 × 10^−16^ (8.76 × 10^−15^)	2.60 (2.06–3.29)	376 (22.3)	3.39 × 10^−5^ (1.86 × 10^−4^)	1.47 (1.23–1.77)	744 (45.0)	3.77 × 10^−44^ (8.29 × 10^−43^)	3.57 (2.99–4.28)
SSc all	467 (326)	115 (12.3)	0.0028 (0.0095)	1.47 (1.14–1.89)	108 (11.6)	0.0035 (0.0095)	1.48 (1.14–1.94)	185 (19.8)	0.0039 (0.0095)	1.25 (1.01–1.54)	174 (26.7)	2.40 × 10^–4^ (0.0011)	1.50 (1.21–1.87)
AAV all	477 (415)	81 (8.5)	0.78 (0.89)	0.96 (0.72–1.27)	84 (8.8)	0.44 (0.76)	1.12 (0.84–1.48)	185 (19.4)	0.083 (0.17)	1.20 (0.98–1.47)	168 (20.2)	0.73 (0.89)	1.04 (0.84–1.28)
Healthy controls	934 (876)	163 (8.7)	referent		149 (8.0)	referent		313 (16.8)	referent		344 (19.6)	referent	

Case-control association analysis was performed using logistic regression test under the additive model using R software. P values (P) and odds ratios (OR) were adjusted for sex. FDR P values (Q) were calculated by Benjamini-Hochberg method. The numbers of the samples in the parentheses show the numbers of those analyzed for rs201802880, after the exclusion of non-amplified samples at the nested PCR process. MAF; minor allele frequency. CI; confidence interval.

Next we performed the association tests of these SNPs with SSc. When compared with healthy controls, the same alleles as in SLE were significantly associated with SSc (Table 1). Among the SNPs, *NCF1* SNP rs201802880 showed the strongest associations with susceptibility to SLE and SSc (SLE: uncorrected P value [P_uncorr_] = 3.77 × 10^−44^, FDR P value [Q] = 8.29 × 10^−43^, Odds Ratio [OR] = 3.57, 95%CI 2.99-4.28; SSc: P_uncorr_ = 2.40 × 10^−4^, Q = 0.0011, OR = 1.50, 95%CI 1.21–1.87, both under the additive model).

In contrast, significant association was not detected in AAV (Table 1). The statistical power to detect association in AAV was calculated to be 51.2% (rs73366469), 48.4% (rs117026326), 73.4% (rs80346167) and 73.4% (rs201802880) for the risk allele with the OR of 1.3.

### Primary role of *NCF1* rs201802880 among the four SNPs

**Figure 1:**
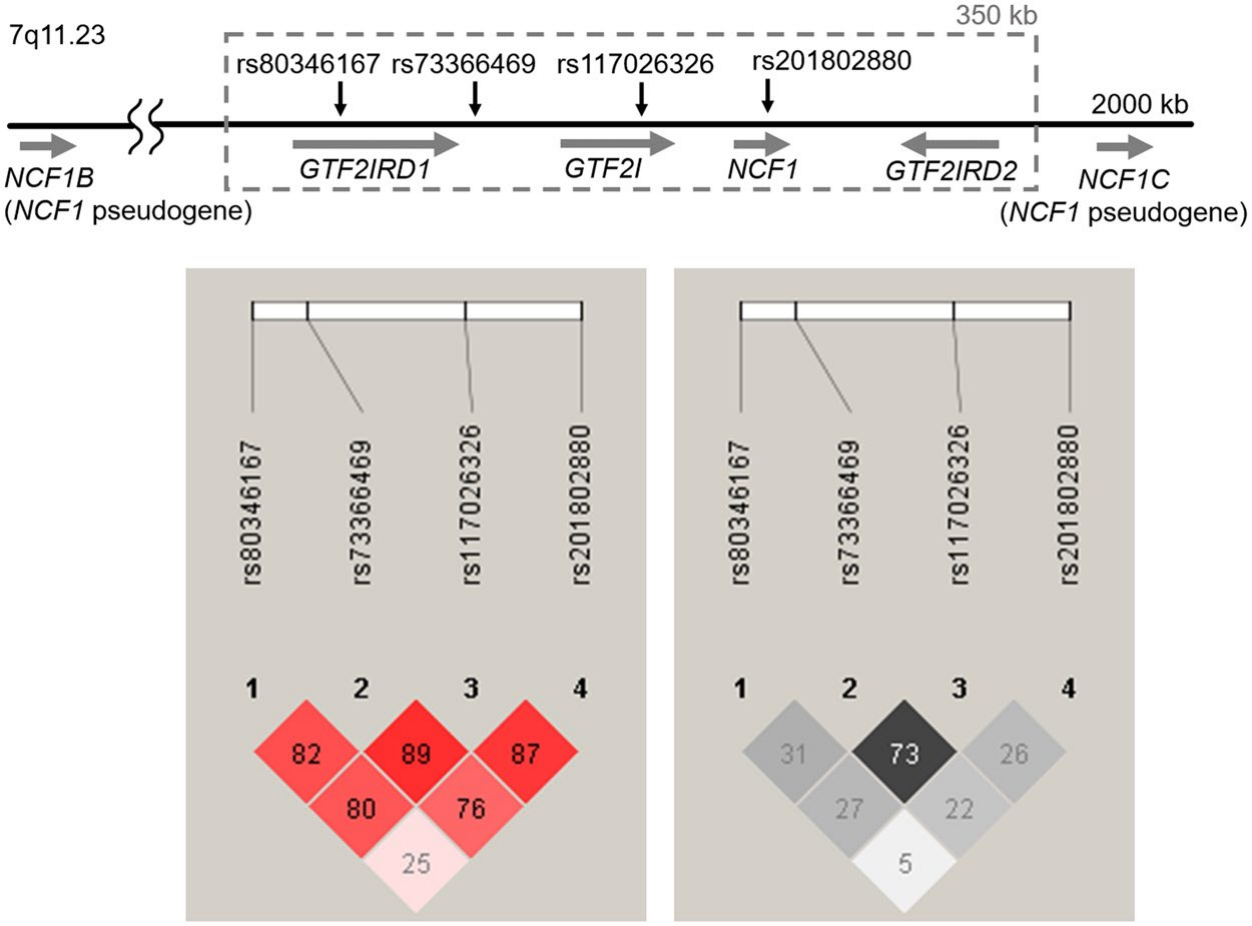
Genomic configuration and linkage disequilibrium (LD) of the SNPs in *GTF2I-NCF1* region. Upper panel shows the genomic configuration of rs73366469 (*GTF2I-GTF2IRD1*), rs117026326 (*GTF2I*), rs80346167 (*GTF2IRD1*) and rs201802880 (*NCF1*). Lower panel; shows the LD plots of the SNPs in 876 Japanese healthy controls of our study (Left: *D’*, Right: *r*^*2*^).

Next we constructed the LD plot of the SNPs of 876 healthy control samples using Haploview 4.2 software. All of the four SNPs were found to be in LD; however, LD between *NCF1* rs201802880 and *GTF2I* SNPs was moderate (Fig. 1).

To determine the primarily associated SNP among the four, conditional logistic regression test was performed with adjustment by each SNP. Notably, the associations of rs201802880 remained significant when conditioned on other SNPs. In contrast, when conditioned on rs201802880, no significant difference remained in other SNPs (Table 2). Therefore, *NCF1* rs201802880 was  considered to be primarily associated with SLE and SSc, while the associations of rs73366469, rs117026326 and rs80346167 were thought to be secondarily caused by LD with rs201802880.

**Table 2:** Primary association of *NCF1* rs201802880 among the *GTF2I-NCF1* region SNPs demonstrated by conditional logistic regression analysis.

		rs73366469		rs117026326		rs80346167		rs201802880	
		P	OR (95%CI)	P	OR (95%CI)	P	OR (95%CI)	P	OR (95%CI)
SLE all	before	4.35 × 10^−14^	2.45 (1.95–3.10)	6.82 × 10^–16^	2.67 (2.11–3.40)	3.54 × 10^−5^	1.48 (1.23–1.79)	3.77 × 10^−44^	3.57 (2.99–4.28)
SSc all	before	0.014	1.45 (1.08–1.95)	0.027	1.41 (1.04–1.92)	0.047	1.28 (1.00–1.59)	2.40 × 10^−4^	1.50 (1.21–1.87)
**Adjusted by rs73366469**									
SLE all	after	—	—	0.0018	2.31 (1.37–2.94)	0.32	0.88 (0.69–1.13)	5.11 × 10^−34^	3.41 (2.81–4.17)
SSc all	after	—	—	0.86	1.06 (0.57–1.95)	0.49	1.11 (0.82–1.49)	0.0043	1.44 (1.12–1.84)
**Adjusted by rs117026326**									
SLE all	after	0.45	1.17 (0.70–1.96)	—	—	0.24	0.87 (0.68–1.10)	2.54 × 10^−32^	3.37 (2.77–4.14)
SSc all	after	0.28	1.39 (0.76–2.50)	—	—	0.35	1.15 (0.86–1.53)	0.0029	1.47 (1.14–1.89)
**Adjusted by rs80346167**									
SLE all	after	5.50 × 10^−11^	2.69 (2.01–3.62)	6.56 × 10^−13^	2.96 (2.21–4.00)	—	—	1.63 × 10^−41^	3.53 (2.95–4.25)
SSc all	after	0.12	1.34 (0.93–1.94)	0.19	1.28 (0.89–1.85)	—	—	9.84 × 10^−4^	1.46 (1.16–1.82)
**Adjusted by rs201802880**									
SLE all	after	0.29	1.15 (0.88–1.51)	0.24	1.18 (0.89–1.56)	0.56	1.06 (0.87–1.30)	—	—
SSc all	after	0.45	1.14 (0.81–1.60)	0.74	1.06 (0.74–1.52)	0.25	1.16 (0.90–1.48)	—	—

Conditional logistic regression analysis was performed under the additive model using R software. P values (P) and odds ratios (OR) were adjusted for sex. P and OR on rows “before” are before adjustment by any other SNPs. P and OR on rows “after” are adjusted by each SNP. In this table, P values are not adjusted for multiple testing. CI; confidence interval.

### Association of *NCF1* rs201802880 with clinical characteristics of SLE and SSc

Finally, we tested whether *NCF1* rs201802880 is associated with specific clinical characteristics of SLE and SSc. Patients with SLE were stratified according to the age of onset ( < 20 years or ≥ 20 years), presence of renal disorders, neurological disorders, anti-dsDNA, anti-Sm and anti-RNP antibodies, and patients with SSc according to diffuse cutaneous SSc (dcSSc) or limited cutaneous SSc (lcSSc), presence or absence of anti-topoisomerase I antibody (ATA), anti-centromere antibody (ACA), and interstitial lung disease (ILD), and case-case analysis was performed. As shown in Table 3, rs201802880 A allele was significantly enriched in the patients with SLE with the age of onset <20 years as compared with the patients with the age of onset ≥ 20 years.

**Table 3:** Association study of *NCF1* rs201802880 and clinical characteristics of SLE and SSc (case-case analysis).

	Additive model for A allele		
	P	Q	OR (95%CI)
**SLE**			
onset < 20 years vs ≥ 20 years	0.0033	0.0095	1.48 (1.14–1.93)
renal disorder present vs absent	0.85	0.89	0.98 (0.80–1.20)
neurologic disorder present vs absent	0.70	0.89	1.05 (0.81–1.36)
anti-dsDNA antibody present vs absent	0.027	0.059	1.35 (1.04–1.77)
anti-Sm antibody present vs absent	0.85	0.89	1.02 (0.82–1.27)
anti-RNP antibody present vs absent	0.80	0.89	1.03 (0.81–1.31)
**SSc**			
dcSSc vs lcSSc	0.45	0.76	0.87 (0.59–1.25)
ATA present vs absent	0.94	0.89	1.01 (0.68–1.49)
ACA present vs absent	0.63	0.89	1.09 (0.77–1.54)
ILD present vs absent	0.65	0.89	1.08 (0.77–1.52)

Genotypes of rs201802880 in SLE and SSc patients with and without specific clinical characteristics were compared using logistic regression analysis under the additive model for A allele with adjustment for sex. Significant enrichment of A allele was observed among SLE patients with onset of <20 years as compared with onset of ≥20 years. FDR P values (Q) were calculated by Benjamini-Hochberg method

dcSSc: diffuse cutaneous SSc, lcSSc: limited cutaneous SSc, ATA: anti-topoisomerase I antibody, ACA: anti-centromere antibody, ILD: interstitial lung disease. Q: FDR P value.

Among the SSc patients, 23 were complicated by RA, SS and/or SLE. Because SLE, SS and RA were already associated with *GTF2I-NCF1* SNPs^2–4,10^, association analysis was also performed after excluding these patients from the SSc group. Significant difference remained after the exclusion of these patients (n = 303, P = 6.58 × 10^−4^, OR = 1.48, 95% CI 1.18–1.85), indicating that the association with SSc did not derive from the patients complicated by SLE, RA and SS.

## Discussion

In this study, *GTF2I-NCF1* region SNPs were strikingly associated with susceptibility to SLE also in the Japanese population. More importantly, the same alleles were found to be associated with susceptibility to SSc for the first time. On the other hand, association was not detected in AAV. Taken together with previous observations on RA^4^ and SS^2^, *GTF2I*-*NCF1* region represents a shared genetic factor for multiple autoimmune rheumatic diseases, but not for AAV.

*NCF1* is located adjacently to *GTF2I* and *GTF2IRD1*, and variants in these genes are in LD. The genomic structure of *NCF1* region is extremely complicated due to presence of two pseudogenes highly homologous to *NCF1*. Two recent studies performed careful association analysis of the *GTF2I*-*NCF1* region with SLE, and reported that a missense mutation in *NCF1*, rs201802880, may be the primarily associated variant in this region^10,11^. Our findings on SLE are consistent with these studies. Taken together with the functional role of *Ncf1* mutation shown by the rodent models^7–9,18^, it is considered that *NCF1* rs201802880 plays a causal role also in human SLE. Although the association of *GTF2I* and *GTF2IRD1* region SNPs reported by GWAS was weaker in the European than in the Asian population, the ORs of *NCF1* rs201802880 were comparable in both populations; thus, the difference in the *GTF2I* associations is likely to be caused by the difference in the LD with *NCF1* between these populations.

The risk allele rs201802880 A (the same allele is denoted as *NCF1* −339T in Olsson *et al*.^11^) was shown to be associated with reduced function of NADPH oxidase, leading to the reduced production of reactive oxygen species (ROS)^11^. Interestingly, the reduced production of ROS has recently been shown to be associated with autoimmune diseases with elevated interferon response in rodents and humans, especially SLE^18^, suggesting a regulatory role of ROS against autoimmunity. The present study also detected that the susceptibility allele rs201802880 A is significantly enriched in SLE patients with younger age of onset, which is consistent with the previous observations in the European population that the age at diagnosis of SLE was significantly younger in the patients carrying the susceptibility allele^10,11^.

On the other hand, lack of association of *GTF2I-NCF1* region with susceptibility to AAV was an unexpected observation, because the role of neutrophil extracellular traps (NETs) has been strongly implicated in AAV as well as in SLE^19^. This lack of association is unlikely to be caused by lack of detection power, because our sample size had 73.4% detection power for a risk allele at *NCF1* rs201802880 with OR of 1.3, and we did not observe even a trend for association. These results suggested that it is unlikely that this allele has substantial genetic contribution to overall AAV, although the possibility that the genetic effect of *NCF1* plays a role in granulomatosis with angiitis (GPA) or proteinase 3-ANCA positive AAV which are rare in the Japanese population cannot be excluded at this point.

In view of the complexity of this genomic region, as well as potential functional relevance of both *GTF2I*/*GTF2IRD1* and *NCF1*, further studies are required to dissect the genetic contribution of this region and to determine whether a single causally associated variation can account for the genetic effect, or multiple variants are independently involved.

In conclusion, the association between *GTF2I-NCF1* region SNPs and susceptibility to SLE was replicated in the Japanese population. In addition, the same alleles were also associated with susceptibility to SSc, but not with AAV. Furthermore, *NCF1* rs201802880 appears to be primarily associated and could account for the genetic associations of other three SNPs. Further studies on *GTF2I-NCF1* region are required to establish the effect size of this shared genetic risk factor among multiple autoimmune rheumatic diseases.

## Methods

### Subjects

Genomic DNA samples from SLE (n = 842, 66 males [7.8%]), SSc (n = 467, 50 males [10.7%]), AAV (n = 477, 190 males [39.8%]) and healthy controls (n = 934, 364 males [39.0%]) were genotyped. All patients and healthy controls are unrelated Japanese, recruited at universities and rheumatology centers in Japan. SLE and SSc patients fulfilled the American College of Rheumatology classification criteria for each disease^20,21^. Presence or absence of renal disorders and neurological disorders in SLE was classified by the same criteria^20^. dcSSc and lcSSc were determined according to the classification criteria by LeRoy *et al*.^22^. The diagnosis of interstitial lung disease (ILD) was made by site investigators based on chest radiography and/or thoracic computed tomography. AAV patients were classified according to the European Medicines Agency (EMEA) algorithm^23^. Autoantibody profiles were determined by ELISA.

### Ethics statement

This study was reviewed and approved by the Ethics Committees of University of Tsukuba, and of the following institutes where the subjects were recruited (in alphabetical order): Aichi Medical University, Asahikawa Medical University, Ehime University, Fukuoka University, Hamamatsu University, Hokkaido University, Hyogo University, Iwate Prefectural Central Hospital, Jichi Medical University, Juntendo University, Kagawa University. Kanazawa University, Kawasaki Municipal Hospital, Kitano Hospital, Kitasato University Hospital, Kobe University Hospital, Kyorin University, Kyoto Prefectural University, Kyoto University, Kyoundo Hospital, Kyushu University, Nagasaki University, Nagoya City University, Nagoya University, National Hospital Organization Kyushu Medical Center, National Hospital Organization Himeji Medical Center, National Hospital Organization Morioka Medical Center, National Hospital Organization Nagoya Medical Center, National Hospital Organization Sagamihara Hospital, National Hospital Organization Shimoshizu National Hospital, Okayama University, Okayama Saiseikai General Hospital, Ome Municipal General Hospital, Saitama Medical Center Hospital, Sendai Shakaihoken Hospital, Shimane University, St. Marianna University, Teikyo University, Tenri Hospital, The University of Miyazaki, The University of Tokyo, Toho University, Tokyo Medical and Dental University, Tokyo Medical University Hachioji Medical Center, Tokyo Metropolitan Komagome Hospital, Tokyo Metropolitan Geriatric Hospital and Institute of Gerontology, Tokyo Metropolitan Tama Medical Center, Tokyo Women’s Medical University, University of Tsukuba, Yokohama City Minato Red Cross Hospital, Yokohama City University Medical Center, Yokohama Minami Kyosai Hospital. This study was conducted in accordance with the principles of the Declaration of Helsinki and the Ethical Guidelines for Human Genome/Gene Analysis Research implemented by Ministry of Education, Culture, Sports, Science and Technology, Ministry of Health, Labour and Welfare, and Ministry of Economy, Trade and Industry, of Japan. Informed consent was obtained from all subjects.

### Nested polymerase chain reaction for the genotyping of rs201802880

Because of the presence of high homologous *NCF1* pseudogenes (*NCF1B* and *NCF1C*), nested polymerase chain reaction (PCR) was employed before TaqMan SNP genotyping assay for rs201802880^10^. An *NCF1*-specific fragment was amplified using KOD FX Neo (TOYOBO, Osaka, Japan) by targeting the GTGT sequence in the exon 2 of *NCF1* (the primer sequences are shown in Supplementary Table 1). The PCR conditions consisted of initial denaturation at 94 °C for 2 min, followed by 35 cycles of denaturation at 98 °C for 10 s, annealing at 60 °C for 30 s and elongation at 68 °C for 2 min. Next, agarose gel electrophoresis was performed to validate the amplification of PCR, and samples without PCR products were excluded. After exclusion, 1μL of the PCR product diluted 1:100 was subjected to TaqMan SNP Genotyping Assay.

### TaqMan SNP genotyping assay

The genotypes of SNPs were determined by TaqMan SNP genotyping assays (ABI 7300, Applied Biosystems). For rs73366469 and rs80346167, the premade primer/probe sets were used (Assay ID: rs73366469: C__97234117_10 and rs80346167: C_100871497_10; Applied Biosystems), and for rs117026326 and rs201802880, the customized primer/probe sets were used (Applied Biosystems, the sequences were shown in Supplementary Table 1). For PCR, DNA samples were added to the reaction mixture containing TaqMan^®^ Genotyping Master Mix (Applied Biosystems) and TaqMan probes. The PCR conditions consisted of initial denaturation at 95 °C for 10 min, followed by 40 cycles (for rs73366469, rs80346167 and rs117026326) or 25cycles (for rs201802880) of denaturation at 95 °C for 15 s, annealing at 60 °C for 60 s.

### Statistical analysis

Association analysis was performed using logistic regression analysis using R software (https://journal.r-project.org) with adjustment for sex. The analysis was performed under the additive, dominant and recessive models (Table 1, Supplementary Tables 2 and 3), and because the Akaike’s Information Criteria (AIC) was the lowest for all SNPs under the additive model in SLE, and almost equal under the three models in SSc and AAV (Supplementary Table 4), the additive model was selected for the association analysis throughout the study. P values for all case-control (Table 1) and case-case analyses (Table 3), 22 comparisons in total, were adjusted for multiple comparisons by controlling false discovery rate (FDR) using Benjamini-Hochberg method^24^. FDR-adjusted P (Q value) < 0.05 was considered significant. Statistical power was calculated by Power and Sample Size Calculation version 3.1.2, 2014 (http://biostat.mc.vanderbilt.edu/wiki/Main/PowerSampleSize)^25^. LD plot was constructed using Haploview 4.2 software (https://www.broadinstitute.org/).

## Supplementary information

Supplementary Tables

## Data Availability

Based on the “Act on the Protection of Personal Inormation” enforced in Japan and the conditions on which the informed consent was given, it is not permitted to disclose an individual’s genotypes and clinical information. All publicly available data generated or analyzed during this study are included in this published article and its Supplementary Information.
