# Supplementary material for: Association of NCF1 polymorphism with systemic lupus erythematosus and systemic sclerosis but not with ANCA-associated vasculitis in a Japanese population
Source: Sci Rep. 2019 Nov 8;9:16366. doi: 10.1038/s41598-019-52920-0 (PMC6842004; doi:10.1038/s41598-019-52920-0)
Supplement: Supplementary file 1 — Supplementary Tables [file 41598_2019_52920_MOESM1_ESM.docx]

**Title:** Association of *NCF1* polymorphism with systemic lupus erythematosus and systemic sclerosis but not with ANCA-associated vasculitis in a Japanese population.

**Authors:**

Nozomi Yokoyama, Aya Kawasaki, Takashi Matsushita, Hiroshi Furukawa, Yuya Kondo, Fumio Hirano, Ken-ei Sada, Isao Matsumoto, Makio Kusaoi, Hirofumi Amano, Shouhei Nagaoka, Keigo Setoguchi, Tatsuo Nagai, Kota Shimada, Shoji Sugii, Atsushi Hashimoto, Toshihiro Matsui, Akira Okamoto, Noriyuki Chiba, Eiichi Suematsu, Shigeru Ohno, Masao Katayama, Kiyoshi Migita, Hajime Kono, Minoru Hasegawa, Shigeto Kobayashi, Hidehiro Yamada, Kenji Nagasaka, Takahiko Sugihara, Kunihiro Yamagata, Shoichi Ozaki, Naoto Tamura, Yoshinari Takasaki, Hiroshi Hashimoto, Hirofumi Makino, Yoshihiro Arimura, Masayoshi Harigai, Shinichi Sato, Takayuki Sumida, Shigeto Tohma, Kazuhiko Takehara, Naoyuki Tsuchiya.

**Supplementary Tables**

Supplementary Table 1. Sequences of the custom primers and probes.

| Target | Primers/Probes | Forward/Reporter 1 | Reverse/Reporter 2 |
| --- | --- | --- | --- |
| rs117026326  (*GTF2I*) | TaqMan Primers | GGTTAGTTTGCATTTTCTATAAAGTCTTATGAATGAAATA | GCTGTGGATGAATTTCAAAACAATCATTT |
|  | TaqMan Probes | CTCCCCGGCCCATG | CTCCCCAGCCCATG |
| rs201802880  (*NCF1*) | Nested PCR Primers | GCTTTCCCCCAGGTGTAC | CCTTCCCTCTCCCACCT |
|  | TaqMan Primers | CAGCTCCCAAGTGGTTTGAC | GGTGGGCAGGCTCATGA |
|  | TaqMan Probes | CCTGGCGGTTCTC | CCTGGTGGTTCTC |

Supplementary Table 2. Associations between the SNPs and SLE, SSc and AAV (dominant model).

|  | n  (n) | rs73366469 (T>C)  (*GTF2I-GTF2IRD1*) | | | | rs117026326 (C>T)  (*GTF2I*) | | | rs80346167 (G>A)  (*GTF2IRD1*) | | | | rs201802880 (G>A)  (*NCF1*) | | |
| --- | --- | --- | --- | --- | --- | --- | --- | --- | --- | --- | --- | --- | --- | --- | --- |
|  |  | MAF  (%) | P | OR  (95%CI) | MAF  (%) | | P | OR  (95%CI) | | MAF  (%) | P | OR  (95%CI) | MAF  (%) | P | OR  (95%CI) |
| SLE all | 842  (826) | 300  (17.8) | 9.00×10^-14^ | 2.52  (1.98-3.23) | 298  (17.7) | | 6.57×10^-16^ | 2.79  (2.18-3.58) | | 376  (22.3) | 1.81×10^-4^ | 1.49  (1.21-1.84) | 744  (45.0) | 6.12×10^-40^ | 4.34  (3.50-5.41) |
| SSc all | 467  (326) | 115  (12.3) | 0.020 | 1.42  (1.06-1.89) | 108  (11.6) | | 0.015 | 1.45  (1.07-1.96) | | 185  (19.8) | 0.17 | 1.18  (0.93-1.51) | 174  (26.7) | 0.0086 | 1.43  (1.09-1.86) |
| AAV all | 477  (415) | 81  (8.5) | 0.97 | 0.99  (0.74-1.33) | 84  (8.8) | | 0.31 | 1.17  (0.87-1.57) | | 185  (19.4) | 0.13 | 1.20  (0.95-1.52) | 168  (20.2) | 0.92 | 0.99  (0.77-1.26) |
| Healthy controls | 934  (876) | 163  (8.7) | referent | | 149  (8.0) | | referent | | | 313  (16.8) | referent | | 344  (19.6) | referent | |

Supplementary Table 3. Associations between the SNPs and SLE, SSc and AAV (recessive model).

|  | n  (n) | rs73366469 (T>C)  (*GTF2I-GTF2IRD1*) | | | | rs117026326 (C>T)  (*GTF2I*) | | | rs80346167 (G>A)  (*GTF2IRD1*) | | | rs201802880 (G>A)  (*NCF1*) | | |
| --- | --- | --- | --- | --- | --- | --- | --- | --- | --- | --- | --- | --- | --- | --- |
|  |  | MAF  (%) | P | OR  (95%CI) | MAF  (%) | | P | OR  (95%CI) | MAF  (%) | P | OR  (95%CI) | MAF  (%) | P | OR  (95%CI) |
| SLE all | 842  (826) | 300  (17.8) | 0.0036 | 3.79  (1.64-10.04) | 298  (17.7) | | 0.0032 | 4.26  (1.73-12.25) | 376  (22.3) | 0.0058 | 2.26  (1.28-4.09) | 744  (45.0) | 4.64×10^-19^ | 7.62  (4.97-12.16) |
| SSc all | 467  (326) | 115  (12.3) | 0.0068 | 3.59  (1.47-9.66) | 108  (11.6) | | 0.010 | 3.71  (1.42-10.88) | 185  (19.8) | 0.0069 | 2.39  (1.27-4.53) | 174  (26.7) | 7.84×10^-5^ | 3.09  (1.77-5.44) |
| AAV all | 477  (415) | 81  (8.5) | 0.23 | 0.28  (0.015-1.58) | 84  (8.8) | | 0.30 | 0.33  (0.017-1.92) | 185  (19.4) | 0.21 | 1.51  (0.78-2.86) | 168  (20.2) | 0.19 | 1.51  (0.80-2.77) |
| Healthy controls | 934  (876) | 163  (8.7) | referent | | 149  (8.0) | | referent | | 313  (16.8) | referent | | 344  (19.6) | referent | |

Supplementary Table 4. Akaike’s Information Criterion (AIC) in the logistic regression analysis of each SNP under additive, dominant and recessive models.

|  | rs73366469 (T>C)  (*GTF2I-GTF2IRD1*) | | | rs117026326 (C>T)  (*GTF2I*) | | | rs80346167 (G>A)  (*GTF2IRD1*) | | | rs201802880 (G>A)  (*NCF1*) | | | |
| --- | --- | --- | --- | --- | --- | --- | --- | --- | --- | --- | --- | --- | --- |
|  | additive  model | dominant  model | recessive  model | additive  model | dominant  model | recessive  model | additive  model | dominant  model | recessive  model | | additive  model | dominant  model | recessive  model |
| SLE all | **2155** | 2157 | 2205 | **2144** | 2146 | 2205 | **2198** | 2201 | 2207 | | **1912** | 1955 | 2031 |
| SSc all | **1673** | 1676 | **1673** | **1673** | 1676 | 1674 | 1677 | 1680 | **1674** | | 1325 | 1332 | **1323** |
| AAV all | 1808 | 1808 | **1806** | 1799 | 1799 | **1798** | **1805** | 1806 | 1807 | | 1623 | 1623 | **1621** |

The lowest AIC in each SNP for each disease is shown in **bold**.
